# Spatiotemporal patterns and ecological factors of tuberculosis notification: A spatial panel data analysis in Guangxi, China

**DOI:** 10.1371/journal.pone.0212051

**Published:** 2019-05-02

**Authors:** Zhezhe Cui, Dingwen Lin, Virasakdi Chongsuvivatwong, Jinming Zhao, Mei Lin, Jing Ou, Jinghua Zhao

**Affiliations:** 1 Department of Tuberculosis Control, Guangxi Zhuang Autonomous Region Center for Disease Control and Prevention, Nanning, Guangxi, China; 2 Epidemiology Unit, Faculty of Medicine, Prince of Songkla University, Songkhla, Thailand; 3 Institute for Communicable Disease Control and Prevention, Qinghai Center for Disease Control and Prevention, Xining, China; The University of Hong Kong, CHINA

## Abstract

**Background:**

Guangxi is one of the provinces having the highest notification rate of tuberculosis in China. However, spatial and temporal patterns and the association between environmental diversity and tuberculosis notification are still unclear.

**Objective:**

To detect the spatiotemporal pattern of tuberculosis notification rates from 2010 to 2016 and its potential association with ecological environmental factors in Guangxi Zhuang autonomous region, China.

**Methods:**

We performed a spatiotemporal analysis with prediction using time series analysis, *Moran’s I* global and local spatial autocorrelation statistics, and space-time scan statistics to detect temporal and spatial clusters of tuberculosis notifications in Guangxi between 2010 and 2016. Spatial panel models were employed to identify potential associating factors.

**Results:**

The number of reported cases peaked in spring and summer and decreased in autumn and winter. The predicted number of reported cases was 49,946 in 2017. *Moran's I* global statistics were greater than 0 (0.363–0.536) during the study period. The most significant hot spots were mainly located in the central area. The eastern area exhibited a low-low relation. By the space-time scanning, the clusters identified were similar to those of the local autocorrelation statistics, and were clustered toward the early part of 2016. Duration of sunshine, per capita gross domestic product, the treatment success rate of tuberculosis and participation rate of the new cooperative medical care insurance scheme in rural areas had a significant negative association with tuberculosis notification rates.

**Conclusion:**

The notification rate of tuberculosis in Guangxi remains high, with the highest notification cluster located in the central region. The notification rate is associated with economic level, treatment success rate and participation in the new cooperative medical care insurance scheme.

## Introduction

Tuberculosis (TB) is a disease caused by the Mycobacterium tuberculosis (MTB) bacteria. Due to the high cost of treatment, the disease imposes a heavy burden on a patient's family and social development [1]. It is a severe public health problem worldwide, especially in developing countries. The burden of TB in China is high. According to the World Health Organization (WHO) [2], the estimated number of TB cases in China was the second highest globally in 2016, after India. Guangxi is an autonomous region located in southern China and is also regarded as a high TB region with about 50,000 new TB cases reported by National Notifiable Disease Reported System (NNDRS) every year. It is ranked as the fifth highest TB burden province in China. The annual notification is about 100 cases per 100,000 population.

Geospatial analytical methods are essential tools for increasing our understanding of public health problems. There is an increasing number of studies that have used geospatial analytical methods to analyze disease trends and to detect the association between the health issues and other factors [3–5]. The TB situation appears complex and spatially heterogeneous in China [6–7]. Previous studies were based on the provincial and city levels [8–9]. Until now, few studies have analyzed the spatiotemporal dynamics of clusters at the county level in China [10]. From our preliminary investigations, notification of cases in Guangxi also exhibits spatial and temporal heterogeneity. Some areas of Guangxi have apparently higher notification rates than other areas. This gap persists even after implementation of active case detection in low notification areas. However, there is a need to quantify these differences using appropriate statistical methods, and to detect similar clusters for special implementation of disease control. In addition, some ecological and environmental factors have been reported as being associated with disease occurrence [11–13]. Diversity of ecological environment in Guangxi indicates that there are possibly some independent variables influencing the spread of the disease [14]. Unfortunately, studies of TB clustering in Guangxi have rarely been reported in the past. Spatial and temporal patterns of TB notification and the association of those clusters are still unclear. For these reasons, we performed a spatiotemporal analysis of TB notifications in Guangxi between 2010 and 2016 using time series analysis, spatial statistical analysis, and space-time scan statistics to detect high and low notification rate clusters. Spatial panel models were employed to identify ecological associative factors.

## Methods

### Data collection

Guangxi has a population of about 46 million. It covers an area of 236,700 km^2^ and has a 1,020 km border with Vietnam to the south-west. Guangxi is divided into 113 counties and districts. Currently, the province has three TB control models. The first is a Center for Disease Control and Prevention (CDC) model covering 51 counties/districts which integrates patient diagnosis, treatment and management into one package. The second is the designated hospital model covering 39 counties/districts which separates patient diagnosis and treatment into the local designated hospital, and patient management into CDC. The third one is a specialist hospital model covering two districts which integrates patient diagnosis, treatment and management into one package in a TB specialist hospital. We collected cases from the NNDRS and those reported by general hospitals, TB designated hospitals, CDC and specialist hospitals, and verified by the National TB Program (NTP) [15]. NNDRS is a multifaceted program that includes a surveillance system for infectious diseases and sharing of data. Public health officers use this information to monitor, control, and prevent the occurrence and spread of nationally notifiable infectious diseases and conditions. The NNDRS records information including name, age, gender, current address, result of sputum test and final diagnosis. TB diagnostic routines include symptoms screening, image examination, sputum smear and culture. If a patient is suspected as high risk for Multiple Drug Resistant Tuberculosis (MDR-TB) then species identification, drug sensitivity testing and Xpert-MTB/RIF assay will be performed. We excluded patients who were diagnosed with non-tuberculosis Mycobacterial (NTM) infection by species identification. Confirmed cases are treated in TB designated hospitals, CDC or specialist hospitals following the directly-observed treatment strategy which includes sustained political and financial commitment, diagnosis by quality ensured sputum-smear microscopy, standardized short-course anti-TB treatment given under direct and supportive observation, an uninterrupted supply of high quality anti-TB drugs and standardized recording and reporting.

Overall, 365,179 TB cases during 2010 and 2016 in Guangxi were reported in this study. We aggregated the cases by county and matched them by area code. Annual population data for each administrative district was obtained from the Sixth Nationwide Population Census database of 2011. Ecological environment data included climatic information, land use, socioeconomic information, health resource and health problems. Table 1 summarizes the classification of ecological environment variables and data sources. After data collection, we constructed a panel data frame with the cross-section of observations repeated over several time periods. We obtained vector map files from the Global Administrative area database (GADM Inc, California, US).

**Table 1:** Classification of ecological environment variables and data sources.

Classification	Variable (abbreviation)	Data sources
Climatic information	Altitude (ALT), Annual rainfall (AR), Duration of sunshine (DOS), Average temperature (AT), Average humidity (AH)	Guangxi Weather Bureau
Land use	Forest cover (FC), Trees in woodland forest (TWF), Trees in sparse forest (TSF), Scattered trees (ST), Trees planted by the side of farm houses, roads, Rivers and fields (TPS)	Guangxi Forestry Bureau
Socioeconomic information	Sex ratio (SR), Total gross domestic product (TGDP), Per capita gross domestic product (PGDP)	Guangxi Yearbook
Health resource	Special funds for TB control (TBF), Health funds (HF), Number of hospitals (NOH), Number of grass-root health facility (NGHF), Number of doctors (NOD), Number of other health workers (NOHW), Participation rate of new cooperative medical care insurance in rural areas (PRR)	Guangxi Health and Family Planning Commission
Health problem	Treatment success rate of TB (TSRTB), Prevalence of HIV/AIDS (PHIV)	Guangxi Health and Family Planning Commission

#### Ethical review

This study was approved by the Institutional Review Board of Guangxi CDC (GW-2017-0001). The ethics committee approved the consent procedure. All participants provided their written informed consent to participate in this study.

### Statistical analysis

#### Time series

The number of TB cases was aggregated by month for trend analysis. We conducted the time series analysis at the provincial level. The time series included 84 months starting from January 2010 and ending in December 2016, and was performed using the *IBM SPSS* statistical data editor (version 19.0, Statistical Product and Service Solutions, Chicago, IL). We used the "forecasting" function to form the sequence chart, and selected the better-predicted model. The predicted model was obtained, together with model diagnostics, and predicted values for the early-warning of TB reporting.

#### Spatial autocorrelation analysis

*Moran’s I* enabled us to measure spatial autocorrelations [16] and has been widely used in the spatial analysis of TB [17–18]. Spatial autocorrelation statistics include global spatial autocorrelation and local spatial autocorrelation. The global spatial autocorrelation estimates the overall degree of dependency among TB notification in a geographic space. The local indicators of spatial association (LISA-cite Anselin) can be used to identify the location and types of clusters. We used GeoDa 1.8.12 (Luc Anselin, University of IL Linois, Urbana-Champaign, US) to analyze the spatial autocorrelation. We also used the empirical Bayes adjustment to take into account variance instability of rates in the global and local spatial autocorrelation.

A Moran’s I value greater than 0 indicates that the notification of TB in neighboring districts exhibits spatial autocorrelation compared to the non-neighboring districts. Notification of districts located close together have similarly high or low rates compared to those located farther away. A value of 0 means that the results may vary slightly due to random permutation. A value less than 0 indicates that there is a negative correlation. Dissimilar values occur near one another [19]. A *P*-value less than 0.05 suggests that we can reject the null hypothesis (notifications are distributed randomly and spatially) and conclude that there are some spatial autocorrelations in the study area. In order to obtain more robust results, we increased the number of permutations to 999. The global Moran’s I statistic is also the mean of the local spatial Moran’s I statistic [16].

*GeoDa* can output the significance map which shows the locations with significant local Moran I statistics in different shades, and a local indicator of spatial association cluster map. The high-high (red districts in the high notification cluster map) and low-low (blue districts in the low notification cluster map) locations are typically regarded to be TB notification hot and cold spots, respectively, while the high-low and low-high locations (negative local spatial autocorrelation) are termed spatial outliers. Outliers are single locations by definition.

#### Space-time scan statistic

In order to test spatial and temporal interaction and evaluate the relative risk for each cluster, we calculated the space-time scan statistics developed by Martin Kulldorff using SaTScan v 7.0.2. For continuous scan statistics, SaTScan uses a continuous Poisson model [20]. The scanning window is an interval (in time), a circle or an ellipse (in space) or a cylinder with a circular or elliptical base. Multiple different window sizes are used. For each location and size of the scanning window, the window with the maximum likelihood is the most likely cluster, that is, the cluster least likely to be due to chance [21]. The alternative hypothesis is that there is an elevated risk within the window as compared to outside. The space-time scan window with maximum likelihood value is defined to be the most likely cluster and other significant windows are defined to be secondary clusters [22]. R v 3.3.2 was used to display the three-dimensional visualization of the scan window.

#### Spatial panel models

The models for spatial panel data were used to estimate the association between the ecological environment factors and notification rate of TB, which could analyze data with spatial dependence and also enable us to consider spatiotemporal heterogeneity. We introduced the spatial panel data models to test the various spatial panel data specifications. Spatial panel data models capture spatial interactions across spatial units and over time. We consider the implementation of maximum likelihood estimators in the context of fixed as well as random effects spatial panel data models according to the following model:

$$y_{it}=\rho \sum_{j}w_{ij}y_{jt}+\beta^{\prime }X_{it}+\alpha_{i}+\gamma_{t}+\varepsilon_{it}$$

where *y_it_* is the annual notification rate of TB. The spatial unit effect, *α*_*i*_ captures unobserved time-invariant heterogeneity and will decrease time-invariant bias from correlated effects. In this section, we used the “splm” package in R to run spatial panel data models. Before running the program, we created a spatial weights matrix according to the neighboring relationship of each location. The object was a list of various elements including the estimated coefficients, the vector of residuals and fitted values. The Baltagi, Song and Koh SLM1 marginal test was employed for checking the random effects [23].

## Results

### Descriptive analysis of TB cases

Of the 365,179 active TB cases collected from NNDRS the annual notifications had clear seasonal fluctuations. The mean annual notification rate of active TB was 113.1/100,000 populations (108.5–117.6). The annual notification rate has fallen by an average of 1.74%. The annual notification rates showed a downtrend (Chi-square for linear trend = 159.76, *P*-value < 0.001).

### Time series analysis

**Fig 1:**
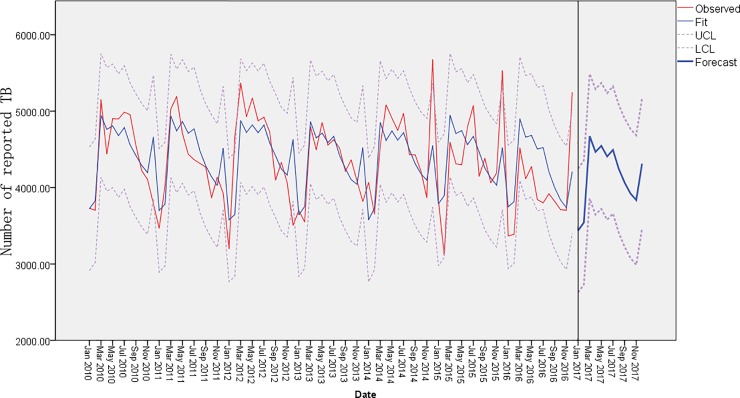
Monthly time series and forecast curve of TB notification in Guangxi, 2010–2017.

Fig 1 shows the monthly time series of active TB cases during 2010–2016 as well as the fitted curves and forecast curves for 2017, including the prediction intervals. The type of forecast model was a simple seasonal model (Stationary *R*^*2*^ = 0.517, normalized *BIC* = 12.121). In general, cases peaked in spring and summer and decreased in autumn and winter. As shown in Table 2, the total forecasted number of reported cases in 2017 was 49,946 with a peak from March to July.

**Table 2:** Forecasted values for tuberculosis notification in 2017.

	Jan	Feb	Mar	Apr	May	Jun	Jul	Aug	Sep	Oct	Nov	Dec	Total
Forecast	3437	3542	4672	4467	4545	4407	4492	4245	4066	3924	3836	4313	49946
Prediction intervals	2628- 4245	2729- 4355	3855- 5488	3646- 5287	3721- 5370	3579- 5236	3660- 5325	3408- 5081	3225- 4906	3079- 4785	2988- 4685	3461- 5165	39980- 59911

### Spatial autocorrelation analysis

As shown in Table 3, Moran's I statistics of every year were greater than 0 (range: 0.363–0.536). During the study period, notification of TB cases in Guangxi exhibited spatial autocorrelation globally.

**Table 3:** Spatial autocorrelation analysis of tuberculosis notification results from 2010 to 2016.

Year	Global spatial autocorrelation	Number of spatial autocorrelation locations				
	*Moran's I Statistic*	high-high	low-low	high-low	low-high	not significant
2010	0.536	19	21	2	1	69
2011	0.476	16	23	2	0	71
2012	0.512	15	25	1	0	71
2013	0.470	12	23	4	1	72
2014	0.520	12	22	2	2	74
2015	0.363	10	24	6	3	69
2016	0.451	15	28	5	3	61

**Fig 2:**
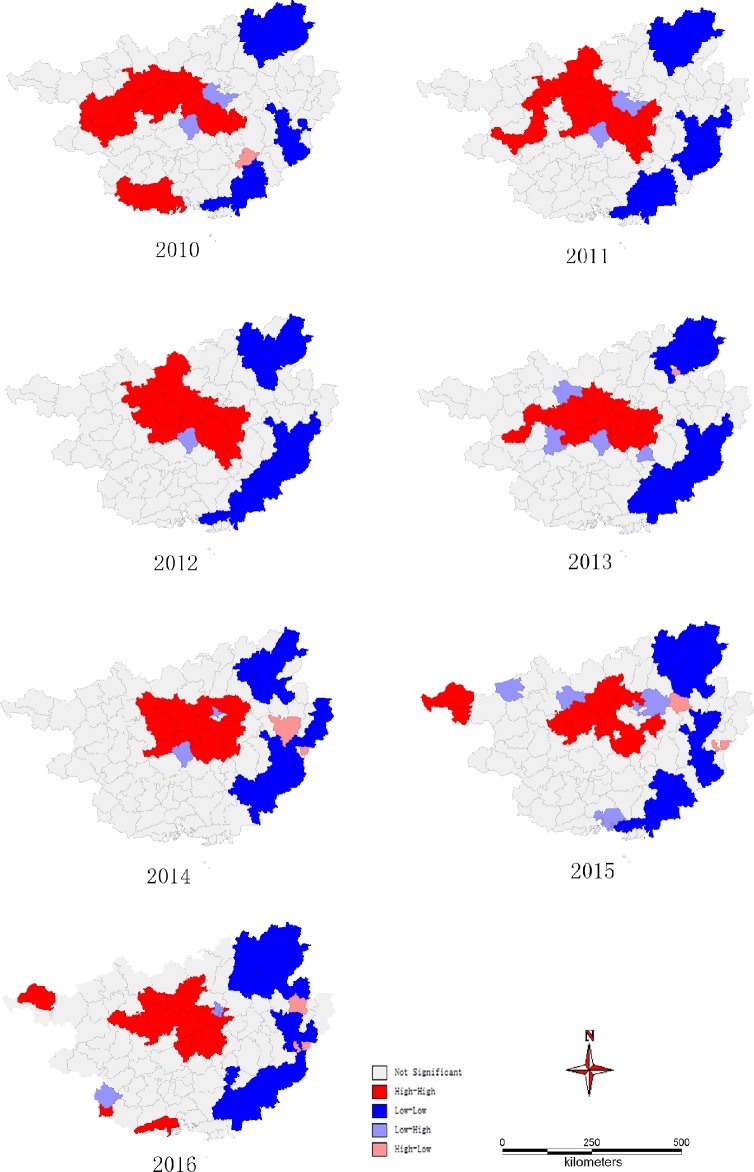
Cluster map for local spatial autocorrelation analysis of TB notification rate in Guangxi, 2010–2016.

From the result of local spatial autocorrelation analysis with empirical Bayes adjustment (Table 3, Fig 2), the most significant hot spots (high-high relation—districts of high notification rates located close to other districts with high notification rates) were mainly located in the central part of Guangxi. Some neighboring districts such as Du’an, Yizhou, Xincheng and Xingbin consistently exhibited a high-high relation as the strongest hot spots during the period of observation. Districts in eastern Guangxi exhibited a low-low relation (significant cold spots—districts with low notification rates located close to other districts with low notification rates). The number of districts exhibiting high-low and low-high correlation were always less than 5. However, the pattern of clustering in some districts changed over the six-year study period. For example, two counties located in the northwest of Guangxi changed from non-significant to significant hot spots, while three counties in the southwest of Guangxi changed from significant hot spots to a non-significant area. The number of districts in significant hot spots decreased from 19 in 2010 to 15 in 2016. In contrast, the number of districts exhibiting significant cold spots in 2016 was more than that in 2010 (28 vs 21).

### Space-time scan statistic

**Fig 3:**
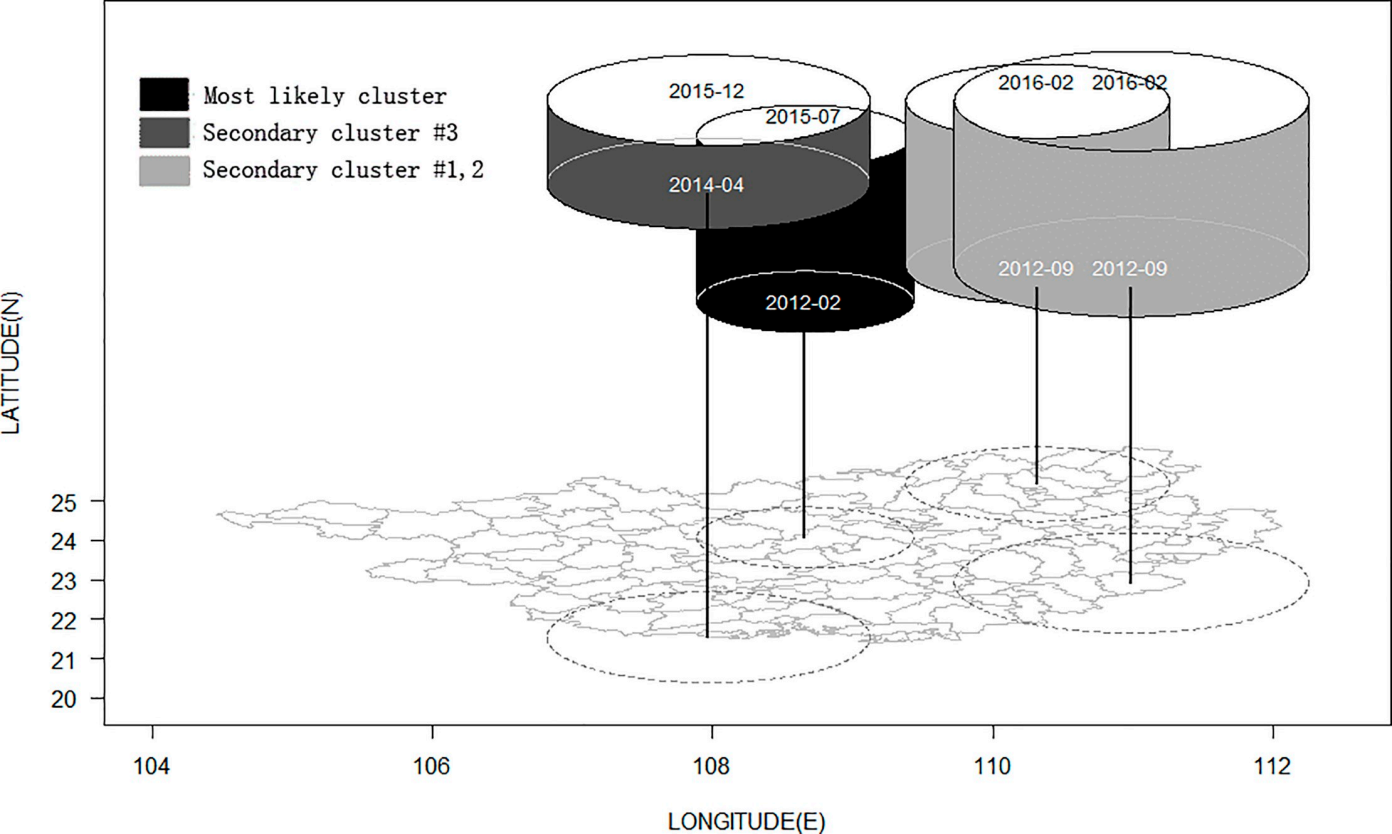
3D cluster window of TB notification based on space-time scan in Guangxi, 2010–2016.

Table 4 shows space-time scan statistics in Guangxi from 2010 to 2016 which considers the spatial and temporal interaction. There was one most likely cluster and three secondary clusters identified. Fig 3 shows that the most likely cluster and secondary cluster #3 were the high notification rate districts (spatiotemporal hot spots) and included 17 counties/districts while the other two clusters were the low notification rate districts (spatiotemporal cold spots) and included 34 counties/districts. As the most likely cluster, the center of the circle is Xincheng county with a radius of 77.3 km, and included nine counties. The cluster period persisted from February 2012 until July 2015. Residents of this region and during this time period had a 1.93 times higher risk of developing active TB compared to those living outside this region. The log likelihood ratio was also very high (4957.7). Another hot spot was located in the south of Guangxi near the Vietnamese border, and persisted from April 2014 until December 2015. The two cold spots were located in the eastern and northern parts of Guangxi, and persisted almost across the whole study period with both having a low relative risk (0.62 and 0.63).

**Table 4:** Space-time clusters of tuberculosis cases in Guangxi from 2010 to 2016.

Cluster	Central point of scan window	Latitude (N)	Longitude (E)	Radius (km)	Cluster period	Number of districts in cluster	Number of cases in cluster	*RR*	*LLR*	*P-value*
Most likely cluster	Xincheng	24.07	108.66	77.32	2012/2/1 to 2015/7/31	9	29846	1.93	4957.7	<0.001
Secondary cluster # 1	Cenxi	22.92	110.99	126.52	2012/9/1 to 2016/2/29	15	27544	0.62	3445.89	<0.001
Secondary cluster # 2	Lingchuan	25.42	110.32	94.1	2012/9/1 to 2016/2/29	17	11965	0.63	1401.91	<0.001
Secondary cluster # 3	Dongxing	21.54	107.97	115.32	2014/4/1 to 2015/12/31	8	11072	1.32	373.81	<0.001

RR: Relative risk; LLR: Log-likelihood ratio

### Spatial panel models

As shown in Table 3, Moran's I values were all greater than 0 between 2010 and 2016. This indicated the existence of high spatial dependency on the notification rate of TB, thus we took the spatial autocorrelation into consideration. Three panel models with spatial lag and spatial error correlation were fitted: an ordinary least squares model, a fixed effects model and a random effects model. The results are shown in Table 5. Spatial autoregressive coefficients (lambda, *λ*) indicate the spatial heterogeneity of variables. By comparing their error variance parameters (rho, *ρ*) as well as checking the random effects [24], we found that the random effects model was better than the other two. The error variance of the random effects model was closest to zero, and the SLM1 marginal test accepted the alternative hypothesis of random effects in the model. This indicates that the intercept term in the random effects model includes the average effect of cross-section random error and time random error, and this model is more suitable for fitting to the data. The duration of sunshine, per capita gross domestic product, the treatment success rate and participation rate of new cooperative medical care insurance in rural areas had a significant negative association with the TB notification rate.

**Table 5:** Results of panel models with spatial lag and spatial error correlation.

Variable/ parameter	Ordinary least squares model		Fixed effects model		Random effects model	
	Estimate	*P*-value	Estimate	*P*-value	Estimate	*P*-value
Altitude	-0.0230	0.0484*	-35.9945	0.0439*	0.0198	0.5463
Annual rainfall	-0.2190	0.0030**	0.0142	0.8108	-0.0244	0.6994
Duration of sunshine	-0.0961	0.0794.	-0.1005	0.0214*	-0.0963	0.0358*
Average temperature	-0.1070	0.4871	0.2208	0.3042	0.3891	0.0592.
Average humidity	-0.0327	0.8523	-0.2010	0.216	-0.0765	0.6542
Forest cover	-0.1085	0.0095**	0.1861	0.3742	-0.0510	0.6329
Trees in woodland Forest	0.0070	0.5992	0.2318	0.0008***	0.0182	0.5265
Trees in sparse forest	0.0117	0.0347*	0.0127	0.0331*	0.0063	0.2937
Scattered trees	0.0004	0.9457	0.0091	0.0605.	0.0059	0.2252
Trees planted by four sides	0.0146	0.0122*	-0.0089	0.1893	-0.0085	0.2085
Sex ratio	-0.4416	0.0373*	0.6122	0.2045	0.0075	0.985
Total gross domestic product	-0.0489	0.0074**	0.0074	0.7583	-0.0028	0.9034
Per capita gross domestic product	-0.1432	<0.0001***	-0.0645	0.0016**	-0.0534	0.0105*
TB control fund	0.0056	0.7048	0.0065	0.5622	0.0107	0.3657
Health fund	-0.0062	0.5191	-0.0128	0.0752.	-0.0073	0.3342
Number of hospitals	-0.0506	0.0221*	0.0181	0.5730	-0.0050	0.8676
Number of grass- root health facility	0.0845	0.0001***	0.1729	0.0141*	0.0702	0.1029
Number of doctors	-0.4139	0.0003***	-0.0029	0.9847	-0.1197	0.4041
Number of other health workers	0.4332	0.0001***	0.0477	0.7222	0.1434	0.284
Treatment success rate of TB	-0.3841	0.0002***	-0.4089	<0.0001***	-0.3940	0.0001***
Prevalence of HIV/AIDS	0.0494	<0.0001***	0.0024	0.8175	0.0150	0.1532
Participation rate of rural areas insurance	0.3884	0.1330	-1.1529	<0.0001***	-0.6083	0.0156*
rho (*ρ*)^a^	-0.7930	<0.0001 ***	-0.7578	<0.0001 ***	-0.7469	<0.0001 ***
lambda (*λ*)^b^	0.7879	<0.0001 ***	0.6384	<0.0001 ***	0.6487	<0.0001 ***
SLM1^c^					30.0490	<0.0001 ***

a: rho, error variance parameter.

b: lambda, spatial autoregressive coefficient.

c: SLM1, Baltagi, Song and Koh SLM1 marginal test for checking random effects.

Significance codes

***, 0.001

**, 0.01

*, 0.05.

## Discussion

This study explored the spatiotemporal pattern of active TB transmission and its ecological association using time series analysis, spatial autocorrelation analysis, space-time scan statistics and spatial panel modeling. The number of active cases reported from the NNDRS is approaching the number estimated by WHO [25]. To our knowledge, this the first study to conduct a spatial panel data analysis of active TB cases in Guangxi, China.

While the global incidence of TB has fallen by an average of about 2% annually between 2000 and 2017 [2], from our study, the notification of active TB cases in Guangxi has had a similar trend. The annual reports of TB cases have remained at a high level with an annual decline rate of 1.74%. The goal of eliminating TB by 2035 will be difficult to achieve unless we take scientific and effective measures to detect TB patients.

The times series of TB in Guangxi showed a seasonal trend over the seven-year period with peaks occurring from March to July. This result is similar to previous studies in Guangxi, Pakistan and Portugal [26–28]. The peaks appeared in spring and summer, a pattern similar to India [29]. Some researchers suggest that most patients who are infected with MTB in the winter then develop active infections during the next 3–6 months [30]. Although about one-third of the population has been infected with TB in the world, many studies from different areas and countries have shown evidence that recent transmission could be the main contributor to epidemics with the development of molecular epidemiology [31–32]. This indicates that a certain percentage of patients have recently been infected with mycobacterium tuberculosis, and developed active disease in a short time. Furthermore, delays in diagnosis and reporting can also lead to the postponement of peaks [10].

In this study, we applied Moran’s I statistics with empirical Bayes adjustment to identify the global and local autocorrelation of cases at the county level. The result of greater than 0 indicated that cases reported by the health department were clustered geographically at the county level. The high and low level of notification exhibit the correlation with each other. Furthermore, it shows high spatial dependency on the notification of TB. The panel models with spatial lag and spatial error correlation maybe an excellent choice [33]. Local patterns of cases display the actual location of high-high relationship (hot spots) and low-low relationship (cold spots). The most significant hot spots were mainly gathered in the central and western parts of Guangxi. Some neighboring districts (Du’an, Yizhou, Xincheng and Xingbin) always exhibited high-high pattern as the strongest hot spots for all seven years. Although the TB prevention department has supplied resource to improve case finding in high TB notification districts, it has little effect on TB control. This inspires us to detect the persistent promotors of the TB transmission.

The data used in this study has two dimensions (location-based variables and timeline). The space-time scan technique which enables researchers to detect the interaction of space and time has been widely used to find spatiotemporal clusters in cancers, including brain cancer [34], lung cancer [35], gastric cancer and breast cancer [36–37], and also infectious diseases such as hand-foot-mouth disease [38,3], dengue fever and tuberculosis [39–40]. A previous study of spatiotemporal clustering characteristics of TB in China showed that the most likely clusters, included Guangxi had a higher TB burden and a higher risk of TB transmission between 2005 and 2011 [8]. By using the space-time scan statistic, we detected one most likely cluster and three secondary clusters in Guangxi between 2010 and 2016 with smaller space and time scales. These clusters were identified similar to findings from the Moran’s I local autocorrelation statistics but with the time variable introduced into the model to detect the temporal clusters. Xincheng county is the central point of the most likely cluster, located in the center of Guangxi with a high relative risk occurring between February 2013 and July 2015. The residents of nine neighboring counties in the most likely cluster had a 1.93 times higher risk of developing active TB compared to those living outside these counties. Compared with the population with high a TB notification rate, the people living in the eastern areas had a lower risk of TB (*RR* = 0.62, Table 3).

Spatial dependence might exist between the outcome and potential impacts at each unit, especially in infectious disease monitoring data [41]. If the variables show spatial dependence, it is appropriate to use the spatial models to detect the influencing factors of observation. Spatial panel models considering the temporal interaction effects between the variables at each spatial unit were commonly used in recent years. In addition, the use of spatial panel model results in more degrees of freedom thus increasing efficiency in the estimation. This study quantified the association using spatial models in Guangxi, and highlights the random effects of spatial panel models to determine the association between TB and ecological environment factors. As our results suggest, a negative relationship between duration of sunshine and TB notification rate was observed, which is consistent with the findings of other recent studies [42–43]. The duration of sunshine in districts with high notification rates was shorter than in districts with low notification rates. This implies that the less someone is exposed to sunshine, the higher the risk of contracting TB. A mechanism for this relationship is that decreased vitamin D levels arising from reduced sunshine exposure impairs the host immune defense system [44]. Once Mycobacterium tuberculosis enters the body, it flourishes, resulting in the occurrence of active tuberculosis. Therefore, it is necessary to encourage people to increase outdoor exercise, especially in the districts with a shorter duration of sunshine. The other significant negative variable is per capita gross domestic product, which is one of the important indexes to measure socioeconomic development. Since poverty was reported as one of the risk factors for TB in the USA [45], it would be problematic if a high TB notification rate correlated with a low-socioeconomic status in the middle of Guangxi. As a logical conclusion based on the study from the USA, low income families tend to live in crowded conditions with scarce health care resources, and these are factors that can facilitate the spread of TB. The growth of economies in eastern Guangxi (TB cold spots) which share a boundary with some counties of neighboring Guangdong province (a more developed province in China) has kept a low level of TB notification. In this study, the lower treatment success rate of TB and inadequate medical insurance coverage led to the long-term existence of the source of infection in a community, which is similar to results of other studies [46–47].

## Limitations

There are some limitations in this study. Firstly, the system was based on passive case findings, a method which was likely to miss some cases. Fortunately, according to the investigation results of the missing rate of infectious diseases, the missing rate of TB notification in Guangxi is less than 5% [48–49]. Patients who do not visit a medical facility should also be considered. Active case finding and advanced diagnostic techniques are two ways to improve TB case detection rates. Secondly, we conducted the space-time scan statistics based on a cylindrical scanning window. Since districts are irregular, this might have led to an erroneous judgment of the number of units in a cluster. Unfortunately, the *Flex-scan* for irregular spatial detecting cannot incorporate time. Thirdly, some potential risk factors such as environmental pollution and socio-economic factors, which may be associated with the TB clustering, were not included in the spatial model. This is due to the lack of monitoring stations at the county level [50–52]. In addition, TB notification in the neighbor cluster may be different due to differences in performance of local TB control programs.

## Conclusion

This study detected a spatial and temporal pattern of TB transmission in Guangxi Zhuang autonomous region using spatio-temporal statistics. The main cluster was located in the central part of Guangxi. Spatial panel modeling with random effects identified that duration of sunshine, per capita gross domestic product, treatment success rate and participation rate of the new cooperative medical care insurance scheme in rural areas had a significant negative association with the TB notification rate.

## Supporting information

S1 File Tuberculosis notification rates and eco-factors panel database of Guangxi, China (2010–2016). (XLSX)
